# Early socioeconomic disadvantage as a predictor of dental care utilisation in adolescence and early adulthood

**DOI:** 10.1186/s12903-026-08049-4

**Published:** 2026-03-19

**Authors:** Amira S Mohamed, Andrea Waylen, Peter G Robinson

**Affiliations:** 1 Bristol Dental School, University of Bristol, Bristol, UK; 2 Faculty of Dentistry, Alexandria University, Alexandria, Egypt

**Keywords:** Socioeconomic Disadvantage, Health Inequities, Dental care, Dental anxiety, Structural Equation Modeling, ALSPAC

## Abstract

**Background:**

Inequalities in dental service utilisation are a universal concern that can exacerbate inequalities in oral health. This study aims to identify the effect of early socioeconomic disadvantage (SED) on dental care utilisation during adolescence and early adulthood.

**Methods:**

Longitudinal secondary analysis of a subsample of the Avon Longitudinal Study of Parents and Children (ALSPAC) using Structural Equation Modelling (SEM). SED was assessed between pregnancy and when the child was two years and nine months. Perceived importance of oral health behaviours, Corah Dental Anxiety Scale, receiving any oral health advice, perceived dental need and the usual reason for dental visits were assessed through a dental questionnaire at 17 years. The effect of early SED and other predictors on the usual reason of dental visits was assessed for those completed the dental questionnaire at 17 years (*n* = 2468) (Model 1). The usual reason of dental visits was also assessed using another questionnaire at 23 years. The effect of early SED and other predictors on the usual reason of dental visits at 23 years was assessed for those completed both questionnaires at 17 and 23 years (*n* = 1639) (Model 2).

**Results:**

Both models showed acceptable fit and yielded similar results. Early SED directly predicted the usual reason for dental visits at 17 years (β= -0.13) and 23 years (β= -0.10). Perceived importance of oral health behaviours was relatively the strongest predictor in both models. Dental anxiety and oral health advice showed indirect effect on irregular dental visits, mediated by high perceived need. High perceived need also mediated the relationship between less perceived importance of oral health behaviours and less regular dental visits.

**Conclusions:**

The results showed the role of early SED. Addressing underlying social inequalities early in life and ensuring universal access to dental care are potential ways to reduce inequalities in dental care utilisation.

**Supplementary Information:**

The online version contains supplementary material available at https://doi.org/10.1186/s12903-026-08049-4.

## Introduction

Inequalities in dental service utilisation are a universal concern that can exacerbate inequalities in oral health [1]. Dental care utilisation is central for the prevention and management of dental diseases [2]. A systematic review of longtudinal studies showed that regular dental visits was associated with better oral health across life in term of less caries experience, less tooth loss and better oral health-related quality of life [3].

Utilisation of dental services is influenced by many factors and is not simply explained by health care characteristics. Differences in adult dental care utilisation and type of visit (preventive or treatment) across 14 European countries persisted after adjusting for oral health needs and the accessibility of facilities [4]. In the UK, the NHS includes a comprehensive dental service free of charge for children until the age of 18 years, students until the age of 19 years, pregnant mothers, unemployed and low-income people and inpatients in hospitals [5]. Despite that, there is still a social gradient in the utilisation of dental care in the UK [6, 7]. A systematic review [2] concluded there were global inequalities in dental visits across gender, ethnicity, immigration status, living place, education, income and insurance coverage.

Andersen’s Behavioural Model of Health Services Use [8, 9] is widely used to understand health and dental care utilisation. Families’ use of health services was predicted by three main components: predisposing factors, enabling factors and the perceived need for treatment.

Social status is a main predisposing factor described by Andersen that identifies the available resources and ability to cope with stressors. Lower levels of education are consistently associated with less dental care utilisation [2, 10]. Low social class was also associated with irregular dental visits among 5–15-year-old children in the UK Children’s Dental Health Survey 2003 [6]. Furthermore, early SED can have a role in dental care utilisation that can be established early and persist across life. Retrospective data on life course dental visits in adults across 13 European countries showed inequalities of regular dental visits based on education started as early as childhood and persisted later in life in most countries [11] The association between dental services use and family income trajectories was assessed based on data from the 1982 Pelotas (Brazil) birth cohort. Individuals who were always poor were less likely to use dental services at 15 and 24 years [12].

Predisposing factors also involve health beliefs that represent knowledge, attitudes and values about health and the use of health services. The inclusion of health beliefs was based on the Health Belief Model (HBM) [13, 14] which was originally developed in the 1950s to understand why people might not participate in preventive programs and to reflect the importance of perceptions of disease susceptibility and effectiveness of treatment.

Enabling factors refer to available resources to obtain health care, such as income and insurance status, the availability of services and having a regular source of care, getting pervious oral health advice and low level of dental anxiety [15, 16]. Higher income was associated with greater use of dental care [17]. Patients who received information about their dental problems were more likely to visit the dentist regularly [18]. Oral health advice received from physicians was also associated with preventive dental visits in children in Western China [19]. Dental anxiety is a common barrier to dental care. It may lead to avoiding or delaying dental visits and complicating oral health problems. Being less dentally anxious predicted better use of dental services [20].

Dental need is a proximal predictor of health care utilisation that has been assessed normatively as the type of dental care needed, such as restorations and extractions [10] or self-determined as perceived oral health status, functional status or need for treatment [16, 21]. Higher need predicts higher utilisation [22].

Most studies on factors affecting utilisation of dental care and visiting the dentist for check-ups have been cross-sectional [17]. Moreover, most used regression methods, whereas techniques such as path analysis and SEM can illuminate the simultaneous relationships between factors [23]. Further research is required using longitudinal studies and representative samples to study the effect of early socioeconomic disadvantage (SED) along with other predisposing factors, enabling factors and need on dental service utilisation [17]. Studying early SED helps to assess early life influences on later health behaviours and outcomes and the need for early interventions. Therefore, this study aimed to identify the effect of early SED on dental care utilisation during adolescence (17 years) and early adulthood (23 years) in a subsample of the Avon Longitudinal Study of Parents and Children (ALSPAC). The study also assessed the effect of perceived importance of oral health behaviours, dental anxiety, receiving oral health advice and perceived need on dental care utilisation and untangled the relationship between them.

## Methods

### Study sample

This study is secondary analysis of data from a birth cohort (ALSPAC). Pregnant women resident in Avon, UK with expected dates of delivery between 1st April 1991 and 31st December 1992 were invited to take part in ALSPAC. The initial number of pregnancies enrolled (known as Phase I enrolment) was 14,541 and 13,988 children were alive at age of one year. The phases of enrolment are described in more detail in the cohort profile paper and its update [24–26].

All ALSPAC participants were invited to focus clinics at different ages. When the mean age of participants was 17.8 years, they were invited to a focus clinic TF4 between December 2008 and June 2011. While 5,214 individuals attended the focus session, 2,643 of those participants completed a self-report dental questionnaire in a paper format. Participants recruited after phase I (did not have data for early SED) and the second child of the twin birth pregnancies were excluded. Data for 2468 participants were analysed to identify the effect of early SED on dental care utilisation in adolescence (17 years).

At approximately 23 years, all ALSPAC participants were invited to complete a questionnaire that included a section about dental health and a question about the usual reason of dental visits. The questionnaire was available to complete in online or paper format between November 2015 and September 2016 and was answered by 4222 participant [27]. Completed paper questionnaires were read using Cardiff TeleForm version 10.1 (Autonomy Corporation plc, Cambridge, England), data collection for the online questionnaires was collected and managed using REDCap electronic data capture tools hosted at the University of Bristol. REDCap (Research Electronic Data Capture) is a secure, web-based software platform designed to support data capture for research studies [28].

**Fig. 1:**
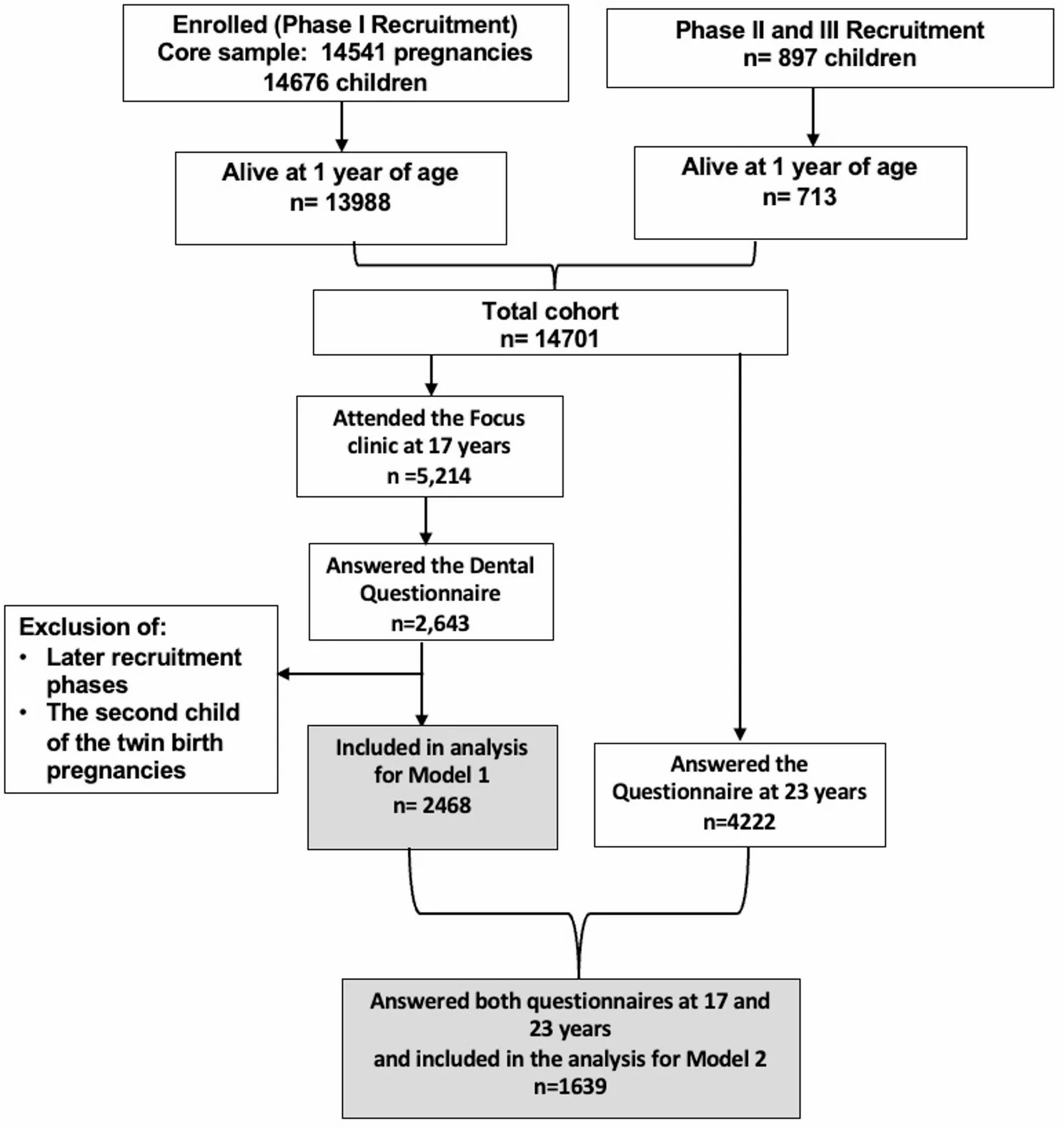
Recruitment of participants to the Avon Longitudinal Study of Parents and Children (ALSPAC) and participants included in the analysis

The effect of early SED on dental care utilisation in early adulthood was assessed for only participants who answered the questionnaires at 17 and 23 years (*n* = 1639). Figure 1 illustrates recruitment of participants to the ALSPAC and numbers of participants included in the analysis.

### Study variables

Potential predictors of dental care utilisation were selected based on the study aim and the best fit for the Andersen framework.

1. Early SED (a predisposing factor) was assessed between pregnancy and two years and nine months through: (a) highest maternal and paternal educational attainment on a five-point ordinal scale (CSE/ none, vocational, O level, A Level and Degree). Data were reverse coded so higher score indicated greater SED, (b) social class based on occupation (previously ‘RGSC’ the Registrar General’s Social Classes) categorised as six social classes [29], (c) Family weekly income was assessed in a self-report questionnaire answered by the mother when the study child was two years and nine months old (1994–1995) (categorized as less than £100, £100–199, £200–299, £300–399, £400 or more). The variable was reverse coded so higher scores indicated greater SED.

The perceived importance of oral health behaviours, enabling factors and need were assessed in a self-report dental questionnaire when participants were aged 17 years.

2) Perceived importance of oral health behaviours (a predisposing factor) was assessed using six questions about the *perceived* importance of six behaviours for dental health (avoiding eating lots of sweets, using fluoride toothpaste, regular dental visits, keeping teeth and gums clean, drinking fluoridated water and using dental floss). Each question had five response options (extremely important, fairly important, doesn’t matter much/not very important, not at all important, do not know). ‘Do not know’ responses were recoded as missing. Answers were reverse-coded, so higher score indicated higher perceived importance of the behaviour.
3) Enabling factors were assessed at 17 years and included: (a) Dental anxiety assessed using the Corah Scale [30] that comprises four questions answered on a five-point scale. The total score ranges from 4 to 20 and higher scores indicate greater dental anxiety. If participants answered that they had never had dental treatment with a drill and had never had their teeth cleaned by dentist, their responses were recoded as missing. The cut off point 13 or more was used to indicate dental anxiety [31]. (b) Getting oral health advice was recorded using a question about the last time the adolescent received oral health advice. The four answer options were: never, in the past year, between one and two years, more than two years. The last three categories were combined due to low prevalence within these categories so that the variable was treated as dichotomous (never, yes).
4) Perceived dental need in adolescence was assessed at 17 years through a question about the adolescent’s belief about treatment needed if they were to visit the dentist tomorrow with five answer options (definitely a lot of treatment is needed, need quite a lot of treatment, need treatment but not much, possibly need some treatment, definitely no treatment needed). Again, this variable was reverse-coded with higher scores indicating greater perceived treatment need.

Dental care utilisation was assessed in dental questionnaire at 17 years and then in a questionnaire at 23 years. The reason why the participant usually went to the dentist was investigated with six answer options: regular routine check-ups (every six or 12 months), occasional check-ups (less than every two years), only when s/he had trouble with their teeth, never go to the dentist, don’t know and another reason. The last two options were recoded as missing and responses were reverse coded so that higher scores indicated more regular and prevention-oriented visits.

### Hypothesised models of dental care utilisation

**Fig. 2:**
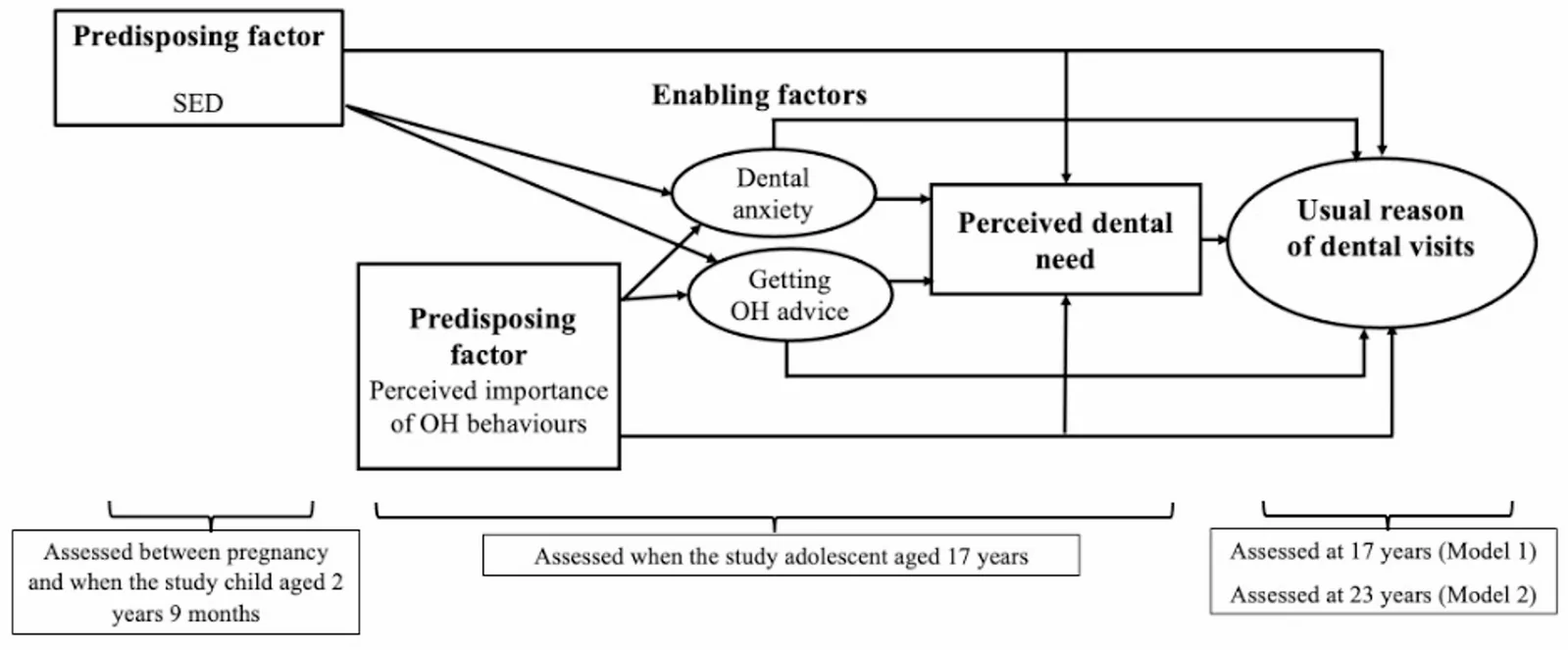
Hypothesised model of dental care utilisation derived from Andersen (1995)

Figure 2 shows the hypothesised model of dental care utilisation, based on an early version of Andersen’s model (1995) and individual level variables [9]. SED early in life was hypothesized to predict less regular dental visits during adolescence and early adulthood either directly or indirectly through enabling factors and perceived treatment need. The model was tested twice for the usual reason for dental visits at 17 years (Model 1) and at 23 years (Model 2).

### Statistical analysis

Descriptive analysis including frequency distributions and measures of central tendency and spread was conducted using STATA^®^ (Version 15.1, StataCorp).

Structural equation modelling was undertaken using Mplus version 8.3 [32] in two stages: (1) Measurement model using exploratory factor analysis (EFA) followed by confirmatory factor analysis (CFA). EFA included all variables potentially measure the underlying constructs (latent variables) to explore factorial structure and assess the strength of relationships between observed variables (indicators) and the underlying construct then the CFA aimed to test the fit of a model with the factors determined in the EFA, (2) Full structural model integrated both the measurement model and the structural model (the hypothesized relationships/pathways between variables) using the mean and variance-adjusted weighted least square (WLSMV). Model fit indices, to assess the model fit, included comparative fit index (CFI) and Tucker-Lewis Index (TLI) with the traditional 0.90 cut off, root mean square error of approximation (RMSEA) and standardized root mean square residual (SRMR), both with < 0.08 cut off [33].

Mediation was also tested using Mplus version 8.3. The direct, total indirect and total effects of the studied factors or variables on dental care utilisation within the studied models were specified. The total indirect effect refers to the sum of one or more indirect pathways between the independent variable and the outcome. This, in addition to the direct effect results in the total effect of this independent variable on the outcome. Bootstrapping was used for less biased standard error and 95% confidence interval (CI) bootstrap percentiles for estimate parameters [34]. The effect size of the direct, indirect and total pathways was considered small if the absolute value of the standardized effect was < 0.1, medium if it was around 0.3 and large if it was > 0.5 [35].

## Results

Data for 2468 adolescents were analysed. Around 39% were males and the mean age (SD) was 17.7 (0.4) and 23.9 (0.5) years while answering the questionnaire at 17 and 23 years respectively. Most adolescents’ parents had at least O level education and were in skilled occupations or higher. More than half of the families had relatively high weekly income (Table 1).

**Table 1:** Socioeconomic characteristics of study participants assessed between pregnancy and two years and nine months (*n* = 2468)

Variables		*n* (%)
Maternal Highest education	Degree	515 (21.5)
	A level	679 (28.3)
	O level	795 (33.2)
	Vocational	177 (7.4)
	CSE	231 (9.6)
Paternal Highest education	Degree	627 (26.9)
	A level	701 (30)
	O level	478 (20.5)
	Vocational	167 (7.2)
	CSE	362 (15.5)
Maternal Social class	I Professional	203 (9.6)
	II Managerial and technical	783 (36.9)
	III Skilled	960 (54.2)
	VI Partly skilled	160 (7.5)
	V Unskilled	15 (0.7)
Parental Social class	I Professional	368 (16.6)
	II Managerial and technical	845 (38.1)
	III Skilled	826 (37.3)
	VI Partly skilled	140 (6.3)
	V Unskilled	39 (1.8)
Family income per week	£400 or more	601 (29.2)
	£300-£399	507 (24.7)
	£200-£299	587 (28.6)
	£100-£199	266 (12.9)
	Less than £100	94 (4.6)

Most adolescents perceived avoiding sweets (79.4%), using fluoride toothpaste (81.5%), maintaining regular dental visits (93.3%) and cleaning teeth (98.5%) as fairly or extremely important. Almost 45% regarded drinking fluoridated water and using dental floss as fairly or extremely important (Table 2).

**Table 2:** Perceived importance of oral health behaviours, enabling factors, perceived need and usual reason of dental visits (*n* = 2468)

Variables		*n* (%)
Perceived importance of avoiding sweets	Not at all or not very important	499 (20.7)
	Fairly or Extremely important	1914 (79.4)
Perceived importance of using fluoride toothpaste	Not at all or not very important	377 (18.5)
	Fairly or Extremely important	1662 (81.5)
Perceived importance of regular dental visits	Not at all or not very important	161 (6.7)
	Fairly or Extremely important	2262 (93.3)
Perceived importance of cleaning teeth	Not at all or not very important	38 (1.5)
	Fairly or Extremely important	2387 (98.5)
Perceived importance of drinking fluoridated water	Not at all or not very important	901(55.2)
	Fairly or Extremely important	732 (44.8)
Perceived importance of using dental floss	Not at all or not very important	1166 (55.5)
	Fairly or Extremely important	935 (44.5)
Getting oral health advice	Yes	1434 (60.3)
Corah Dental anxiety Scale	Mean (SD)	7.70 (3.16)
Perceived treatment need	Definitely no treatment needed	864 (35.6)
	possibly need some treatment	1260 (51.9)
	Yes, but not much	257 (10.6)
	Yes, need quite a lot	36 (1.5)
	Definitely a lot of treatment needed	11(0.5)
	Very poor	21 (0.9)
Usual reason of dental visit at 17 years	Never go to dentist	6 (0.2)
	Only when has trouble	61 (2.5)
	Occasional check-up (< every 2 y)	154 (6.3)
	Regular routine check-up (every 6–12 m)	2207 (90.9)
Usual reason of dental visit at 23 years (*n* = 1639)	Never go to dentist	36 (2.2)
	Only when has trouble	175 (10.8)
	Occasional check-up (< every 2y)	254 (15.7)
	Regular routine check-up (every 6–12 m)	1154 (71.3)

Around 60% of the participants had received previous oral health advice and only 8.2% of the participants were dentally anxious. The mean Corah dental anxiety Scale (SD) was 7.7 (3.16). Nearly half of the adolescents perceived possible need for dental treatment. Around 91% usually went to the dentist for regular routine check-ups (every 6 or 12 months) at 17 years while 71.3% went for regular routine check-ups (every 6 or 12 months) at 23 years (Table 2).

EFA was conducted followed by CFA. The final measurement model included two factors: SED and perceived importance of oral health behaviours and showed a good fit (RMSEA = 0.053, CFI = 0.960, TLI = 0.950, SRMR = 0.043). Father education was the best indicator for early SED (β = 0.81) followed by mother education (β = 0.78). Perceived importance of keeping teeth and gums clean was the best indicator of perceived importance of oral health behaviours (β = 0.78) followed by perceived importance of regular dental visits (β = 0.68). A figure demonstrating the measurement model is provided in Suppl 1.

**Fig. 3:**
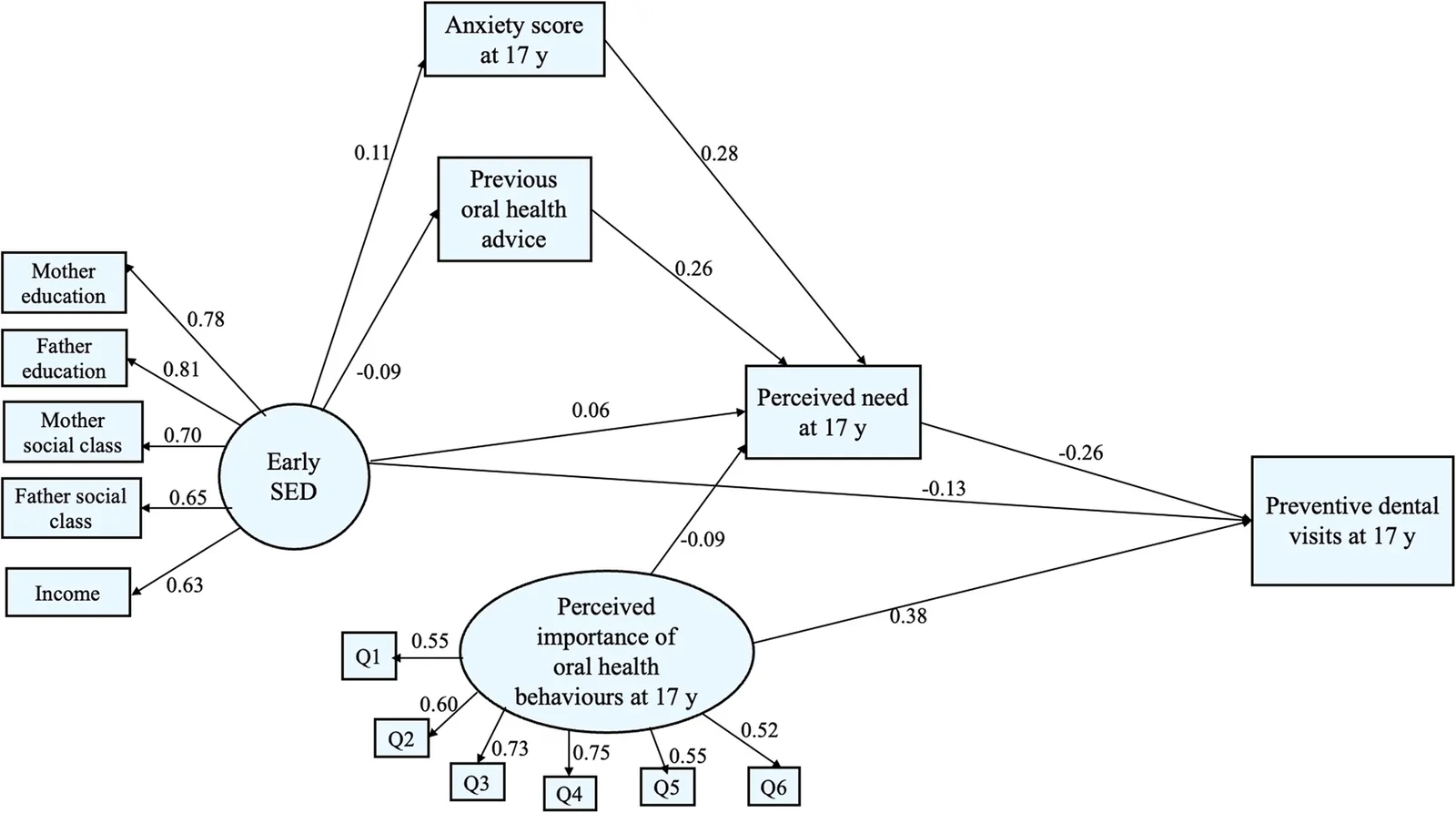
Standardised estimates for SEM of usual reason for dental visits at 17 years (Model 1). β bootstrapped standardised estimates are shown. bootstrapped standard error ranged from 0.01 to 0.05, Only significant pathways are shown

In the full structural model, there was little evidence of an association between perceived importance of oral health behaviours and dental anxiety or receipt of oral health advice so these pathways were removed from the model to improve fit. The final model showed acceptable fit (RMSEA = 0.054, CFI = 0.945, TLI = 0.930, SRMR = 0.053) and explained 26.4% of the variance in the usual reason for a dental visit at 17 years (Model 1) (Fig. 3). The model showed that adolescents who were socio-economically disadvantaged early in life were less likely to receive oral health advice (β=-0.09), were more dentally anxious (β = 0.11), perceived higher dental need (β = 0.06) and have less preventive visits (β= -0.13). Perceiving oral health behaviours as important was associated with perceiving less dental need (β=-0.09) and more preventive visits (β = 0.38). On the other hand, dental anxiety and receiving oral health advice were both associated with higher perceived dental need (β = 0.28, β = 0.26 respectively). Those who perceived their dental need as high were less likely to have preventive visits (β= -0.26). 

Mediation analysis tested the direct, indirect and total effects of model components on the usual reason for dental visits at 17 years (Table 3). Early SED directly predicted less regular dental visits at 17 years (β= -0.13). Perceived importance of oral health behaviours was positively associated with regular dental visits directly (β = 0.38) and indirectly (β = 0.02) through less perceived dental need. Being dentally anxious or getting any previous oral health advice were indirectly associated with irregular dental visits mediated by a higher perceived need with same standardised effect size (β=-0.07).

**Table 3:** Standardized effects of early SED and other model components on the usual reason for dental visits at 17 years (*n* = 2468)

Effect on the usual reason for dental visit at 17 years			β	Bootstrap SE	95%CI Bias corrected	*p*-value
Early SED	Total		-0.16*	0.04	-0.24, -0.07	< 0.001
	Direct		-0.13*	0.04	-0.22, -0.05	0.003
	Total indirect		-0.02	0.01	-0.05, 0.01	0.089
	Indirect pathways	Oral health advice	-0.004	0.01	-0.02, 0.01	0.464
		Dental anxiety	-0.01	0.00	-0.01, 0.00	0.237
		Perceived importance of oral health behaviours	0.00	0.01	-0.02, 0.03	0.853
		Perceived dental need	-0.02*	0.01	-0.03, -0.00	0.031
		Oral health advice- Perceived dental need	0.01*	0.00	0.00 ,0.01	0.013
		Dental anxiety -Perceived dental need	-0.01*	0.00	-0.01, -0.00	0.001
		Perceived importance of oral health behaviours- Perceived dental need	0.00	0.00	-0.00, 0.01	0.856
Perceived importance of Oral health behaviours	Total		0.41*	0.04	0.34, 0.48	< 0.001
	Direct		0.38*	0.04	0.31, 0.46	< 0.001
	Total indirect		0.02*	0.01	0.01, 0.04	0.004
	Indirect pathways	Perceived dental need	0.02*	0.01	0.01, 0.04	0.004
Dental anxiety	Total		-0.12*	0.04	-0.19, -0.05	0.001
	Direct		-0.05	0.04	-0.12, 0.03	0.216
	Total indirect		-0.07*	0.02	-0.10, -0.05	< 0.001
	Indirect pathway	Perceived dental need	-0.07*	0.02	-0.10, -0.05	< 0.001
Oral health advice	Total		-0.03	0.05	-0.12, 0.06	0.534
	Direct		0.04	0.05	-0.05, 0.13	0.407
	Total indirect		-0.07*	0.01	-0.10, -0.04	< 0.001
	Indirect pathway	Perceived dental need	-0.07*	0.01	-0.10, -0.04	< 0.001
Perceived need	Direct		-0.26*	0.05	-0.35, -0.17	< 0.001

* *p* < 0.05, β bootstrapped standardised estimate, *SE* standard error, *CI* confidence interval

**Fig. 4:**
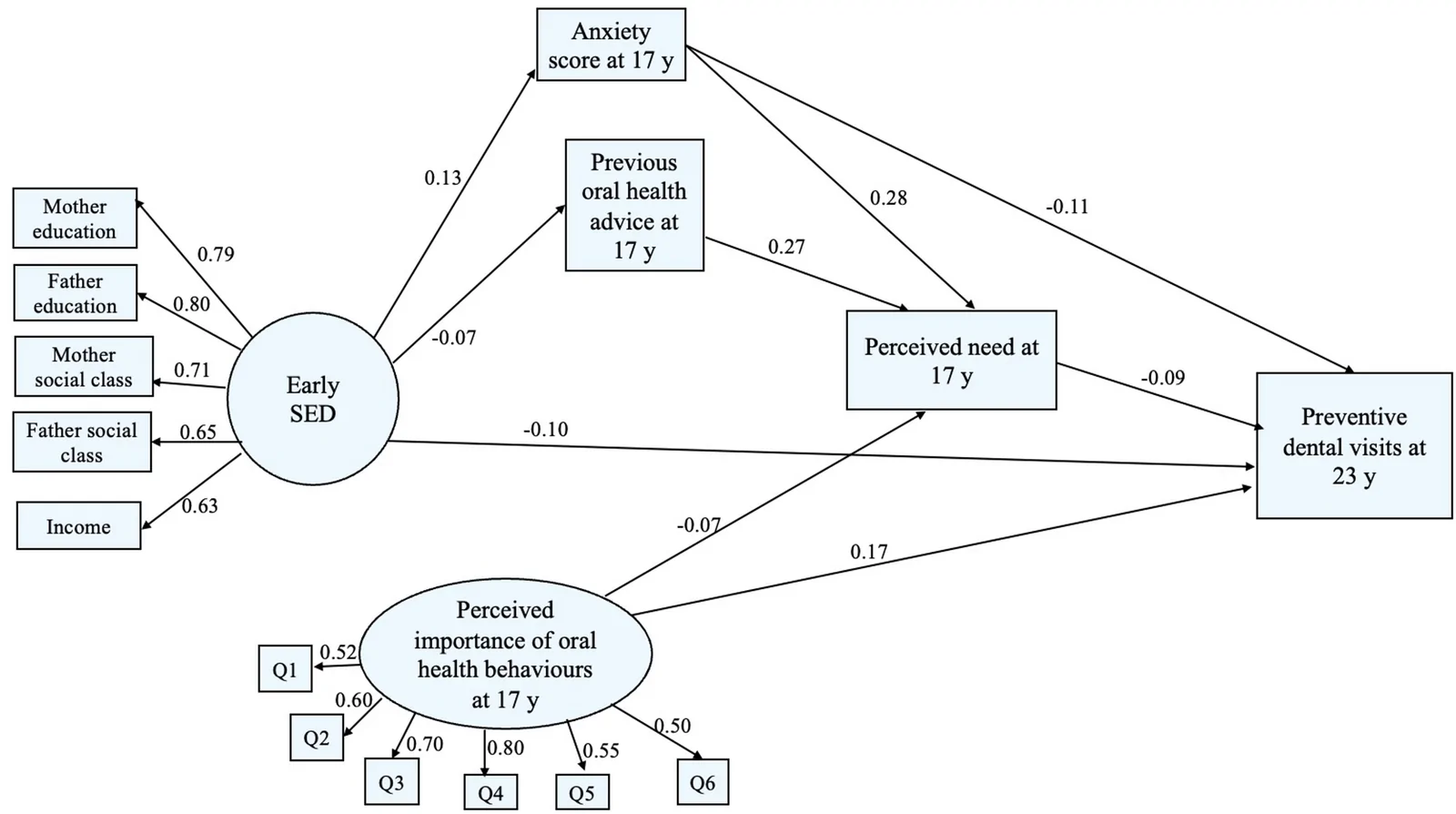
Standardised estimates for SEM of usual reason for dental visits at 23 years (Model 2) β bootstrapped standardised estimates are shown. bootstrapped standard error ranged from 0.01 to 0.05, Only significant pathways are shown

The model for usual reason for dental visits at 23 years (Model 2) showed acceptable fit (RMSEA = 0.045, CFI = 0.960, TLI = 0.949, SRMR = 0.043). The bootstrapped standardized estimates are shown in Fig. 4. The model explained 7.1% of the variance in the usual reason for a dental visit. Model 2 yielded compatible results with Model 1. Standardized effects size were generally lower than those in model 1. Unlike model 1, dental anxiety showed a negative direct effect on regular dental visits at 23 years (β= -0.11) (Table 4).

**Table 4:** Standardized effects of early SED and other model components on the usual reason for dental visits at 23 years (*n* = 1639)

Effect on the usual reason for dental visit at 23 years			β	Bootstrap SE	95%CI Bias corrected	*p*-value
Early SED	Total		-0.12*	0.04	-0.19, -0.06	< 0.001
	Direct		-0.10*	0.04	-0.17, -0.03	0.004
	Total indirect		-0.02	0.01	-0.04, -0.00	0.056
	Indirect pathways	Oral health advice	-0.00	0.00	-0.01, 0.01	0.850
		Dental anxiety	-0.02*	0.01	-0.03, -0.00	0.014
		Perceived importance of oral health behaviours	0.00	0.01	-0.01, 0.02	0.679
		Perceived dental need	-0.01	0.00	-0.02, 0.00	0.185
		Oral health advice - Perceived dental need	0.00	0.00	0.00, 0.01	0.169
		Dental anxiety -Perceived dental need	-0.00	0.00	-0.01, 0.00	0.063
		Perceived importance of oral health behaviours- Perceived dental need	0.00	0.00	0.00, 0.00	0.726
Perceived importance of oral health behaviours	Total		0.17*	0.04	0.10, 0.25	< 0.001
	Direct		0.17*	0.04	0.09, 0.24	< 0.001
	Total indirect		0.01	0.00	0.00, 0.02	0.154
	Indirect pathway	Perceived dental need	0.01	0.00	0.00, 0.02	0.154
Dental anxiety	Total		-0.14*	0.04	-0.21, -0.07	< 0.001
	Direct		-0.11*	0.04	-0.18, -0.04	0.002
	Total indirect		-0.03*	0.01	-0.05, -0.00	0.03
	Indirect pathway	Perceived dental need	-0.03*	0.01	-0.05, -0.00	0.03
Oral health advice	Total		-0.02	0.04	-0.10, 0.06	0.713
	Direct		0.01	0.04	-0.08, 0.09	0.833
	Total indirect		-0.02*	0.01	-0.05, -0.00	0.029
	Indirect path	Perceived dental need	-0.02*	0.01	-0.05, -0.00	0.029
Perceived need	Direct		-0.09*	0.04	-0.17, -0.01	0.022

* *p* < 0.05, β bootstrapped standardised estimate, *SE* standard error, *CI* confidence interval

Model 2 was tested again while including usual reason for dental visits at 17 years as a predictor for usual reason for dental visits at 23 years (Suppl II). The model showed acceptable fit (RMSEA = 0.049, CFI = 0.949, TLI = 0.933, SRMR = 0.052). The model mainly showed that preventive visits at 23 years was directly predicted by preventive visits at 17 years (β = 0.39) and dental anxiety (β= -0.09). Effect of early SED on preventive visits at 23 years was indirect via preventive dental visits at 17 years (β= -0.06). Similarly, effect of perceived importance of oral health was indirect (β = 0.15). Anxiety also showed indirect effect on usual reason for dental visits at 23 years mediated by dental need then the usual reason for dental visits at 17 years (β= -0.03).

## Discussion

This study aimed to assess the effect of early SED on the usual reason of the dental visit during adolescence and early adulthood. Early SED predicted less regular dental visits during adolescence and same finding was obtained during early adulthood. These findings support the critical/sensitive period model of health inequalities [36]. While the critical/sensitive model mainly explain oral health inequalities, this study focused on inequities in dental care utilisation that can in turn affect oral health. The study also found that perceiving oral health behaviours as important predict regular dental visits while having dental anxiety can predict irregular dental visits.

Most of the sample visited the dentist for routine check-ups during adolescence; this might be related to the affluent characteristics of the sample, favourable beliefs about preventive oral health behaviours and dental care being provided to adolescents at 17 years at no charge within the NHS. Nevertheless, inequalities in dental care utilisation were still found, which are in line with the British Household Panel Survey among adults aged 16 years and over in UK over the period 1991–2008 [7]. This agrees with a previous study in Poland showing inequalities in dental service utilisation although free dental care is available to children [37].

This study is among few assessing dental care utilisation based on birth cohorts. The inequalities shown in this study builds on another study using data from the 1982 Pelotas (Brazil) investigating the effect of income trajectories on oral health behaviours at 15 and 24 years [12]. Income was assessed at three time points (birth, 15 and 23 years) while our study focused on the impact of early SED. In contrast to our study, a cross-sectional study based on the 1982 Pelotas birth cohort did not show an association between use of dental services and income or education at 31 years old that was attributed to sample size or characteristics [38].

The effect of SED on dental care utilisation was mainly direct or may have been mediated by unstudied variables such as locus of control, sense of coherence, registration with a dentist and perceptions of the dental service as accessible and affordable.

Testing the model for dental care utilisation during early adulthood yielded similar findings to that during adolescence. Differences between both models can be related to changes in dental visiting behaviour toward early adulthood with leaving full time education and subsidised dental care. It is noteworthy that the explanatory power of model 2 and effect sizes for early SED and other model components on usual reason of dental visits may have been further attenuated, due to dilution of early life influences and being more independent. Attenuation may also be attributed to greater influence of unstudied enabling factors such as insurance coverage in adulthood adding additional variation.

Perceived importance of oral health behaviours was relatively the strongest predictor of regular dental visits at 17 and 23 years. Model 1 showed both direct effect and indirect effects through less perceived dental need. These health beliefs about the relevance of oral health behaviours such as tooth brushing and decreasing sugars could lead to better oral health and less dental problems so dental visits were asymptomatic rather than therapeutic. The role of perceived importance of oral health behaviours in predicting dental visits is in agreement with other studies [39–41]. This supports the incorporation of the health belief model into the Andersen model for explaining utilisation of dental services. The relatively low importance placed on water fluoridation by participants may reflect that most of them lived in a non-fluoridated area and would have little control over this variable. It may be counter intuitive that perceived importance of oral health behaviours was not predicted by early SED,. This be due to sample homogeneity, most of whom showed favourable oral health beliefs and affluent characteristics. Perceived importance of oral health behaviours can be shaped during adolescence through different exposures and socialization with dilution of early SED impact. Dental anxiety was associated with less regular visits at 17 years, mediated by higher dental need (Model 1). This can be related to dental visits avoidance that increase dental need [15, 20]. Furthermore, dental anxiety directly predicted less regular visits at 23 years (Model 2). There was not enough evidence for this direct pathway of dental anxiety at 17 years, which may be due to less sample variability with regards to regular dental visits at 17 years. While the effect size of most of the other variables decreased toward adulthood, only dental anxiety showed higher total and direct effects that reflect the impact of dental anxiety that can trace through life stages. Unlike other studies that found oral health advice to be positively associated with regular visits [15, 18, 19], previous advice was associated with less regular visits at 17 and 23 years via an indirect pathway mediated by higher perceived dental need. Oral health advice may increase awareness of oral health problems and may have increased the perception of treatment need. Oral health advice might have been received due to oral health concerns or problems in the first place that implies a potential reverse causality between getting advice and perceived treatment need, which is possible as both variables were collected at the same time (17 years). The evidence of direct and total effect of getting any previous oral health advice was still insufficient. While the Andersen model suggests a positive relationship between need and utilisation, adolescents who perceived their dental need as high at 17 years were less likely to attend for regular visits at 17 years. The same negative association was found between perceived dental need assessed at 17 years and regular visits at 23 years (Model 2). This ruled out potential reverse causality in Model 1 where both variables assessed at 17 years. The negative association between dental need and regular dental visits agrees with other studies [10, 15, 42]. The relationship between need and utilisation is inconsistent due to differences in the variables used, ranging from clinical status to perceived health problems and the potential for reverse causality in cross-sectional studies.

This study used data from a population-based ALSPAC. The birth cohort longitudinal design, over more than 20 years gave better evidence for the temporal relation between variables assessed at three stages through the life course: pregnancy to infancy, adolescence and early adulthood. It minimized recall bias especially with the long time between the exposure and the outcomes. It also minimized the potential reverse causality associated with cross-sectional studies investigating early life influences. Assessment of dental care utilization at different time points allowed testing the model for dental care utilization at 17 years then at 23 years and compare findings. Structural equation modelling of the hypothesised model helped to assess the direct and indirect effects of predictors of dental care utilisation simultaneously.

There are some limitations related to this secondary analysis. Data on relevant enabling factors such as registration with the dentist, perceived access to dental service and psychological factors were not available. The sample was relatively affluent and attended for regular dental visits, especially at 17 years. Such homogeneity might have affected the evidence for effect of some variables so further studies in deprived communities are required. The dataset is relatively old, but it is likely that similar relationships persist between variables. The study still gives insights to early life influences and factors affecting dental care utilisation.

Early interventions for improving utilisation of dental care are recommended. Interventions to target inequalities in dental care utilization in adulthood rather than across the life course may be ineffective [11]. Minimizing social inequalities in populations and improving access to dental care are needed. Although access to oral health care was not tested in this study, most participants reported regular routine check-up (91%) at 17 years in comparison to 71% at 23 years that might be related to eligibility for NHS dental treatment only at 17 years. This can indicate the role of improving access to oral health care in decreasing inequalities in regular dental visits in affluent population. It also agrees with data from 11 European countries showing less inequalities in countries with dental health care coverage in comparison to countries with no public coverage [43]. In addition, community based approaches can employ non-dental health personal to provide support and encourage regular dental visits.

Early management of dental anxiety and dental needs can also encourage regular dental visits and in turn promote oral health.

## Conclusions

This study showed inequalities in dental care utilization and highlighted the relevance of early life factors on oral health behaviours later in life. The study also showed perceived importance of oral health behaviours and dental anxiety as predictors of the usual reason for dental visit during adolescence and early adulthood.

## Supplementary Information

Supplementary Material 1.

## Data Availability

The ALSPAC website contains details of all the data that is available through a fully searchable data dictionary and variable search tool (http:/www.bristol.ac.uk/alspac/researchers/our-data/).

## Abbreviations

SED: socioeconomic disadvantage
ALSPAC: Avon Longitudinal Study of Parents and Children

## References

[1] Goswami S, Tseveenjav B, Kaila M. Non-utilization of oral health services and associated factors among children and adolescents: an integrative review. Acta Odontol Scand. 2023;81(2):105–18. 10.1080/00016357.2022.2095020 35841154

[2] Reda SF, Reda SM, Thomson WM, Schwendicke F. Inequality in utilization of dental services: a systematic review and meta-analysis. Am J Public Health. 2018;108(2):e1–7. 10.2105/AJPH.2017.304180 PMC5846590 29267052

[3] Mohd Khairuddin AN, Bogale B, Kang J, Gallagher JE. Impact of dental visiting patterns on oral health: A systematic review of longitudinal studies. BDJ open. 2024;10(1):18. 10.1038/s41405-024-00195-7 PMC10917741 38448428

[4] Listl S, Moran V, Maurer J, Faggion CM Jr. Dental service utilization by Europeans aged 50 plus. Commun Dent Oral Epidemiol. 2012;40(2):164–74. 10.1111/j.1600-0528.2011.00639.x 21895735

[5] Garla BK, Satish G, Divya K. Dental insurance: A systematic review. J Int Soc Prev Community Dentistry. 2014;4(Suppl 2):S73. 10.4103/2231-0762.146200 PMC4278106 25558454

[6] Shaban R, Kassim S, Sabbah W. Socioeconomic inequality in the provision of specific preventive dental interventions among children in the UK: Children’s Dental Health Survey 2003. Br Dent J. 2017;222(11):865–9. 10.1038/sj.bdj.2017.499 28703180

[7] Vernekar N, Batchelor P, Heilmann A. Adult self-reported attendance for dental check-ups over a 16-year period in the UK. Br Dent J. 2019;226(11):883–8. 10.1038/s41415-019-0366-8 31203343

[8] Andersen RM. Families’ use of health services: a behavioral model of predisposing, enabling, and need components. Purdue University; 1968. https://docs.lib.purdue.edu/dissertations/AAI6902884.

[9] Andersen RM. Revisiting the behavioral model and access to medical care: does it matter? J Health Soc Behav. 1995;36:1–10. 7738325

[10] Vargas CM, Ronzio CR. Relationship between children’s dental needs and dental care utilization: United States, 1988–1994. Am J Public Health. 2002;92(11):1816–21. 10.2105/ajph.92.11.1816 PMC1447334 12406814

[11] Listl S. Inequalities in dental attendance throughout the life-course. J Dent Res. 2012;91(7suppl):S91–7. 10.1177/0022034512447953 PMC3383102 22699676

[12] Peres KG, Peres MA, Demarco FF, Tarquínio SBC, Horta BL, Gigante DP. Oral health studies in the 1982 Pelotas (Brazil) birth cohort: methodology and principal results at 15 and 24 years of age. Cadernos de saude Publica. 2011;27:1569–80. 10.1590/s0102-311x2011000800012 21877005

[13] Hochbaum GM. Public participation in medical screening programs: a socio-psychological study. Los Angeles: US Department of Health, Education, and Welfare, Public Health Service; 1958.

[14] Rosenstock IM. Historical origins of the health belief model. Health Educ Monogr. 1974;2(4):328–35. 10.1177/109019817800600406 299611

[15] Baker S. Applying Andersen’s behavioural model to oral health: what are the contextual factors shaping perceived oral health outcomes? Commun Dent Oral Epidemiol. 2009;37(6):485–94. 10.1111/j.1600-0528.2009.00495.x 19845712

[16] Herkrath FJ, Vettore MV, Werneck GL. Utilisation of dental services by Brazilian adults in rural and urban areas: a multi-group structural equation analysis using the Andersen behavioural model. BMC Public Health. 2020;20(1):1–13. 10.1186/s12889-020-09100-x PMC7301519 32552777

[17] Hajek A, Kretzler B, König H-H. Factors associated with dental service use based on the Andersen model: a systematic review. Int J Environ Res Public Health. 2021;18(5):2491. 10.3390/ijerph18052491 PMC7967618 33802430

[18] Quadri FA, Jafari FA, Albeshri AT, Zailai AM. Factors influencing patients’ utilization of dental health services in Jazan, Kingdom of Saudi Arabia. Int J Clin Pediatr dentistry. 2018;11(1):29. 10.5005/jp-journals-10005-1479 PMC5968159 29805231

[19] Qu X, Houser SH, Tian M, Zhang Q, Pan J, Zhang W. Effects of early preventive dental visits and its associations with dental caries experience: a cross-sectional study. BMC Oral Health. 2022;22(1):1–9. 10.1186/s12903-022-02190-6 PMC9052678 35488264

[20] Armfield JM, Stewart JF, Spencer AJ. The vicious cycle of dental fear: exploring the interplay between oral health, service utilization and dental fear. BMC Oral Health. 2007;7(1):1–15. 10.1186/1472-6831-7-1 PMC1784087 17222356

[21] Bell JF, Huebner CE, Reed SC. Oral health need and access to dental services: evidence from the National Survey of Children’s Health, 2007. Matern Child Health J. 2012;16(1):27–34. 10.1007/s10995-012-0992-0 22456986

[22] Al Agili DE, Farsi NJ. Need for dental care drives utilisation of dental services among children in Saudi Arabia. Int Dent J. 2020;70(3):183–92. 10.1111/idj.12539 PMC9379191 31912900

[23] Babitsch B, Gohl D, Von Lengerke T. Re-revisiting Andersen’s Behavioral Model of Health Services Use: a systematic review of studies from 1998–2011. GMS Psychosoc Med. 9:Doc11. http://www.egms.de/en/journals/psm/2012-9/psm000089.shtml. 10.3205/psm000089 PMC3488807 23133505

[24] Boyd A, Golding J, Macleod J, Lawlor DA, Fraser A, Henderson J, et al. Cohort profile: the ‘children of the 90s’—the index offspring of the Avon Longitudinal Study of Parents and Children. Int J Epidemiol. 2013;42(1):111–27. 10.1093/ije/dys064 PMC3600618 22507743

[25] Fraser A, Macdonald-Wallis C, Tilling K, Boyd A, Golding J, Davey Smith G, et al. Cohort profile: the Avon Longitudinal Study of Parents and Children: ALSPAC mothers cohort. Int J Epidemiol. 2013;42(1):97–110. 10.1093/ije/dys066 PMC3600619 22507742

[26] Northstone K, Lewcock M, Groom A, Boyd A, Macleod J, Timpson N, et al. The Avon Longitudinal Study of Parents and Children (ALSPAC): an update on the enrolled sample of index children in 2019. Wellcome open Res. 2019;4:51. 10.12688/wellcomeopenres.15132.1 PMC6464058 31020050

[27] Dudding T, Haworth S, Sandy J, Timpson NJ. Age 23 years+ oral health questionnaire in Avon longitudinal study of parents and children. Wellcome Open Res. 2018;3:34. 10.12688/wellcomeopenres.14159.2 PMC5941244 29806037

[28] Harris PA, Taylor R, Thielke R, Payne J, Gonzalez N, Conde JG. Research electronic data capture (REDCap)—a metadata-driven methodology and workflow process for providing translational research informatics support. J Biomed Inform. 2009;42(2):377–81. 10.1016/j.jbi.2008.08.010 PMC2700030 18929686

[29] Pevalin D, Rose D. The national statistics socio-economic classification: unifying official and sociological approaches to the conceptualisation and measurement of social class in the United Kingdom. Sociétés contemporaines. 2002;1:75–106.

[30] Corah NL. Development of a dental anxiety scale. J Dent Res. 1969;48(4):596. 10.1177/00220345690480041801 5256508

[31] Humphris G, Freeman R, Campbell J, Tuutti H, D’souza V. Further evidence for the reliability and validity of the Modified Dental Anxiety Scale. Int Dent J. 2000;50(6):367–70. 10.1111/j.1875-595x.2000.tb00570.x 11197195

[32] Muthẽn L, Muthẽn B. Mplus version 8 user’s guide (Version 8). Los Angeles, CA: Muthẽn & Muthẽn; 2017.

[33] Wang J, Wang X. Structural equation modeling: applications using Mplus. 2nd ed. New York, NY, USA: Wiley; 2019.

[34] Shrout PE, Bolger NJP. Mediation in experimental and nonexperimental studies: new procedures and recommendations. 2002;7(4):422. 12530702

[35] Cohen J. Statistical power analysis for the behavioural sciences. Hillsdale (NJ):Lawrence Erlbaum Associates; 1988.

[36] Ben-Shlomo Y, Kuh D. A life course approach to chronic disease epidemiology: conceptual models, empirical challenges and interdisciplinary perspectives. Int J Epidemiol. 2002;31:285–93. 11980781

[37] Opydo-Szymaczek J, Borysewicz-Lewicka M, Andrysiak K, Witkowska Z, Hoffmann-Przybylska A, Przybylski P, et al. Clinical consequences of dental caries, parents’ perception of child’s oral health and attitudes towards dental visits in a population of 7-year-old children. Int J Environ Res Public Health. 2021;18(11):5844. 10.3390/ijerph18115844 PMC8198085 34072416

[38] Borges RC, Echeverria MS, Karam SA, Horta BL, Demarco FF. Use of dental services among adults from a birth cohort in the South region of Brazil. Rev Saude Publica. 2023;57:47. 10.11606/s1518-8787.2023057004604 PMC10392773 37585946

[39] Telleen S, Rhee Kim YO, Chavez N, Barrett RE, Hall W, Gajendra S. Access to oral health services for urban low-income Latino children: social ecological influences. J Public Health Dent. 2012;72(1):8–18. 10.1111/j.1752-7325.2011.00275.x 22316105

[40] Broadbent J, Zeng J, Foster Page L, Baker S, Ramrakha S, Thomson W. Oral health–related beliefs, behaviors, and outcomes through the life course. J Dent Res. 2016;95(7):808–13. 10.1177/0022034516634663 PMC4914862 26936215

[41] Lee CY, Ting CC, Wu JH, Lee KT, Chen HS, Chang YY. Dental visiting behaviours among primary schoolchildren: Application of the health belief model. Int J Dental Hygiene. 2018;16(2):e88–95. 10.1111/idh.12319 28984068

[42] Marshman Z, Porritt J, Dyer T, Wyborn C, Godson J, Baker S. What influences the use of dental services by adults in the UK? Commun Dent Oral Epidemiol. 2012;40(4):306–14. 10.1111/j.1600-0528.2012.00675.x 22409397

[43] Palència L, Espelt A, Cornejo-Ovalle M, Borrell C. Socioeconomic inequalities in the use of dental care services in Europe: what is the role of public coverage? Commun Dent Oral Epidemiol. 2014;42(2):97–105. 10.1111/cdoe.12056 PMC3864569 23786417

