# Supplementary material for: Early socioeconomic disadvantage as a predictor of dental care utilisation in adolescence and early adulthood
Source: BMC Oral Health. 2026 Mar 19;26:772. doi: 10.1186/s12903-026-08049-4 (PMC13130465; doi:10.1186/s12903-026-08049-4)
Supplement: Supplementary file 1 — Supplementary Material 1. [file 12903_2026_8049_MOESM1_ESM.docx]

Supplemental material I


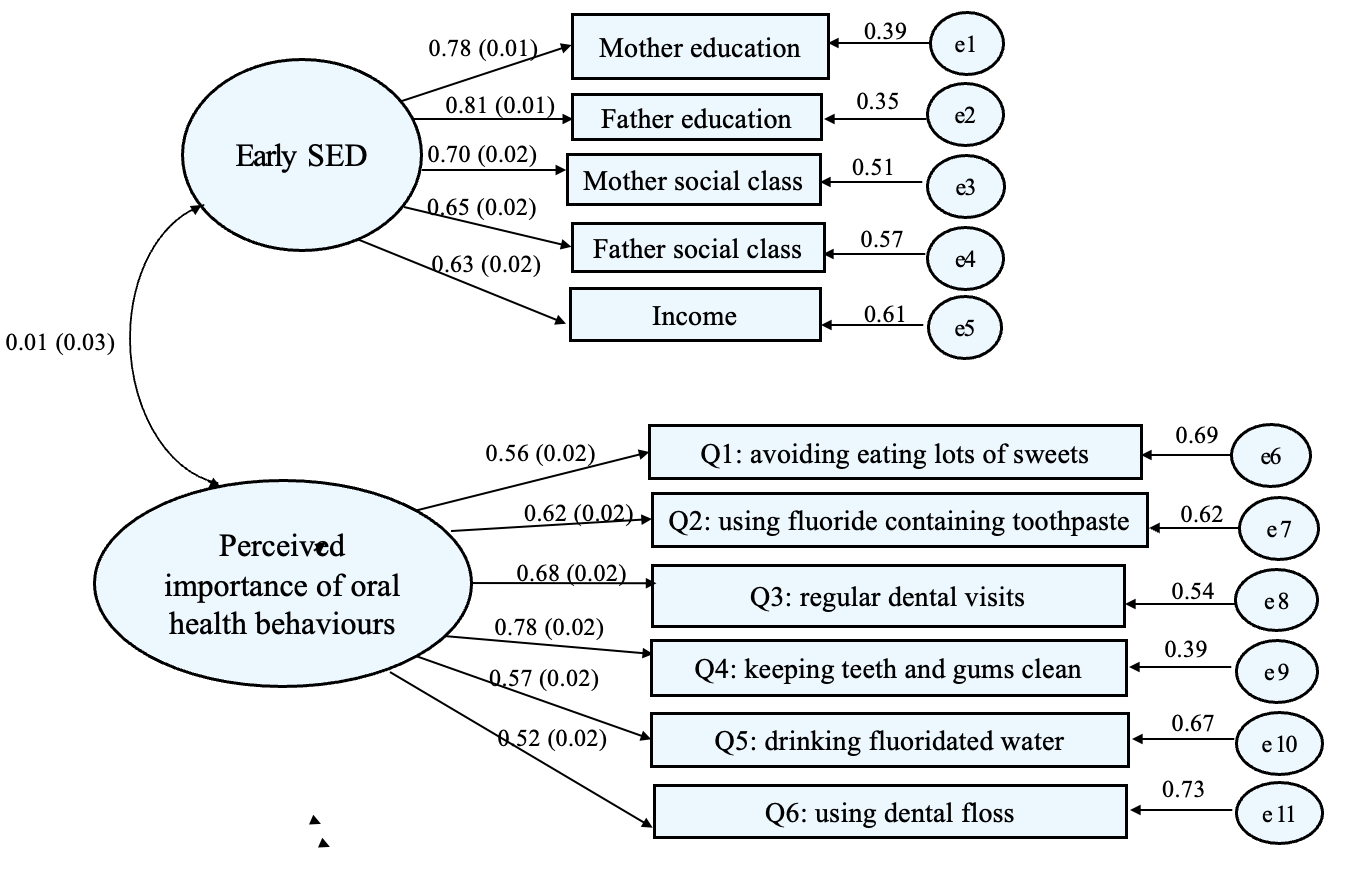


Figure showing measurement model

Ellipses represent latent factors while rectangles represent observed variables, single-headed straight arrows represent a regression path while the curved arrows represent covariances or correlations.

Supplemental material II


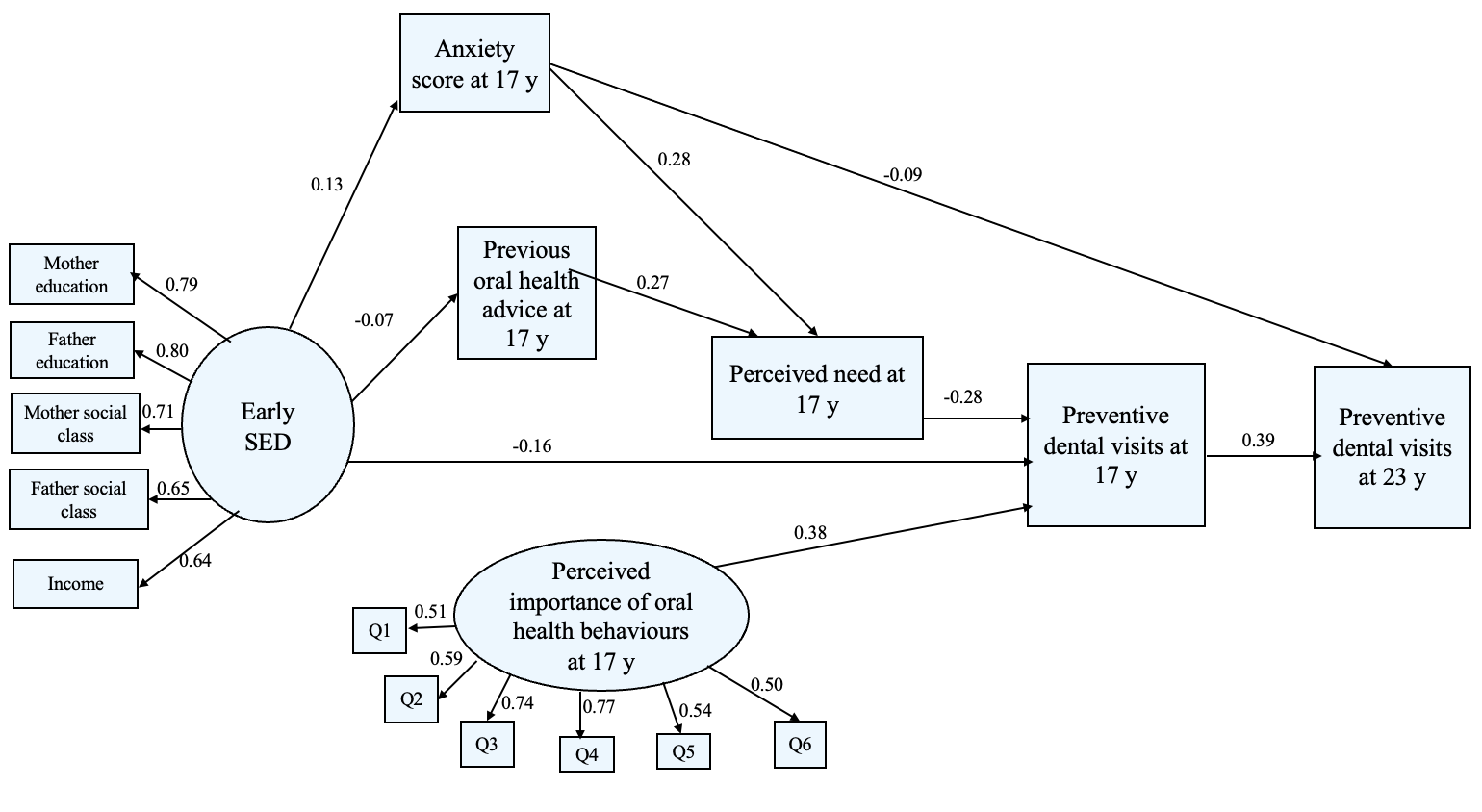


**Table. Standardized effects of early SED, preventive dental visits at 17 years and other model components on the usual reason for dental visits at 23 years (n=1639).**

| **Effect on the usual reason for dental visit at 23 years** | | | **β** | **Bootstrap SE** | **95%CI Bias corrected** | **p-value** |
| --- | --- | --- | --- | --- | --- | --- |
| **Early SED** | Total | | -0.12* | 0.04 | -0.19, -0.06 | 0.001 |
|  | Direct | | -0.04 | 0.04 | -0.11, 0.04 | 0.307 |
|  | Total indirect | | -0.08* | 0.03 | -0.15, -0.04 | 0.005 |
|  | Indirect pathways (only pathways with p ≤0.05 are shown) | Preventive dental visits at 17 years | -0.06* | 0.03 | -0.13, -0.02 | 0.022 |
|  |  | Dental anxiety | -0.01* | 0.01 | -0.02, -0.001 | 0.038 |
|  |  | Dental anxiety - Perceived dental need- Preventive dental visits at 17 years | -0.004* | 0.00 | -0.01, -0.00 | 0.027 |
| **Perceived importance of oral health behaviours** | Total | | 0.18* | 0.04 | 0.11, 0.25 | <0.001 |
|  | Direct | | 0.02 | 0.05 | -0.08, 0.11 | 0.641 |
|  | Total indirect | | 0.15* | 0.04 | 0.10, 0.24 | <0.001 |
|  | Indirect pathway with with p ≤0.05 | Preventive dental visits at 17 years | 0.15* | 0.04 | 0.09, 0.23 | <0.001 |
| **Dental anxiety** | Total | | -0.14* | 0.04 | -0.21, -0.07 | <0.001 |
|  | Direct | | -0.09* | 0.04 | -0.17, -0.01 | 0.019 |
|  | Total indirect | | -0.05* | 0.02 | -0.09, 0.00 | 0.04 |
|  | Indirect pathway with with p ≤0.05 | Perceived dental need- Preventive dental visits at 17 years | -0.03* | 0.01 | -0.05, -0.01 | 0.004 |
| **Oral health Advice** | Total | | -0.02 | 0.04 | -0.09, 0.06 | 0.708 |
|  | Direct | | -0.02 | 0.05 | -0.12, 0.07 | 0.740 |
|  | Total indirect | | 0.00 | 0.03 | -0.05, 0.06 | 0.982 |
|  | Indirect path with with p ≤0.05 | Perceived dental need- Preventive dental visits at 17 years | -0.03* | 0.01 | -0.05, -0.01 | 0.005 |
| **Perceived need** | Total | | -0.09* | 0.04 | -0.16, -0.01 | 0.024 |
|  | Direct | | 0.02 | 0.05 | -0.06, 0.11 | 0.704 |
|  | Total indirect | | -0.11* | 0.04 | -0.18, -0.05 | 0.003 |
|  | Preventive dental visits at 17 years | | -0.11* | 0.04 | -0.18, -0.05 | 0.003 |
| **Preventive dental visits at 17 years** | Direct | | 0.39* | 0.07 | 0.27, 0.54 | <0.001 |

* p < 0.05, β bootstrapped standardised estimate, SE standard error, CI confidence interval
